# Diffusion-weighted MRI and histogram analysis: assessment of response to neoadjuvant chemotherapy in nephroblastoma

**DOI:** 10.1007/s00261-021-03032-9

**Published:** 2021-03-12

**Authors:** Andreas M. Hötker, Yousef Mazaheri, André Lollert, Jens-Peter Schenk, Junting Zheng, Marinela Capanu, Oguz Akin, Norbert Graf, Gundula Staatz

**Affiliations:** 1 Institute of Diagnostic and Interventional Radiology, University Hospital Zurich, Rämistrasse 100, 8091 Zurich, Switzerland; 2 Department of Medical Physics, Memorial Sloan Kettering Cancer Center, 1275 York Avenue, New York, NY 10065 USA; 3 Department of Radiology, Universitätsmedizin Mainz, Langenbeckstr. 1, 55131 Mainz, Germany; 4 Division of Pediatric Radiology, University Hospital Heidelberg, Im Neuenheimer Feld 153, 69120 Heidelberg, Germany; 5 Department of Epidemiology and Biostatistics, Memorial Sloan Kettering Cancer Center, 1275 York Avenue, New York, NY 10065 USA; 6 Department of Radiology, Memorial Sloan Kettering Cancer Center, 1275 York Avenue, New York, NY 10065 USA; 7 Department of Pediatric Oncology and Hematology, Saarland University, Kirrberger Straße, 66421 Homburg, Germany

**Keywords:** Nephroblastoma, Magnetic resonance imaging, Diffusion-weighted imaging

## Abstract

**Purpose:**

To assess the value of diffusion-weighted MRI (DW-MRI) in the non-invasive prediction of blastemal remnant after neoadjuvant chemotherapy in nephroblastoma.

**Methods:**

This IRB-approved study included 32 pediatric patients with 35 tumors who underwent DW-MRI prior and after completion of neoadjuvant chemotherapy and subsequent surgical resection. Two blinded radiologists volumetrically assessed each tumor on pre- and post-neoadjuvant images and the parameters mean ADC, median ADC, 12.5th/25th/75th ADC percentile, skewness, and kurtosis were calculated. Blastemal remnant was determined per the pathology report. Associations between imaging features and blastemal remnant quartiles were examined using the Kruskal–Wallis test and adjusted for false discovery rate.

**Results:**

Inter-reader agreement was high for mean ADC, skewness, kurtosis, and volume (ICC: 0.76–0.998). Pre-therapeutic histogram parameters skewness and kurtosis were found to be higher in patients with a higher amount of blastemal remnant for reader 1 (overall *p* = 0.035) and for kurtosis in reader 2 (overall *p* = 0.032) with skewness not reaching the level of statistical significance (overall *p* = 0.055). Higher tumor volume on pre-treatment imaging was associated with a higher amount of blastemal remnant after therapy (overall *p* = 0.032 for both readers).

**Conclusions:**

Pre-treatment skewness and kurtosis of ADC histogram analysis were significantly associated with a larger fraction of a blastemal remnant after neoadjuvant chemotherapy. These findings could be incorporated into a more personalized chemotherapeutic regime in these patients and offer prognostic information at the time of initial diagnosis.

**Supplementary Information:**

The online version contains supplementary material available at 10.1007/s00261-021-03032-9.

## Introduction

Nephroblastoma is the most common renal malignancy in children [1]. In Europe, according to International Society of Pediatric Oncology (SIOP) guidelines, the treatment for nephroblastoma includes neoadjuvant chemotherapy (commonly without prior biopsy) followed by surgical resection of the tumor [2]. Several markers indicating a poor prognosis have been identified in patients with nephroblastoma, including higher tumor stage, older age at time of diagnosis, as well as several specific molecular markers (e.g., loss of heterozygosity of 1p and 16q, and gain of 1q) that are commonly associated with a higher risk of relapse and mortality. Viable blastemal remnant in the tumor after neoadjuvant treatment is a marker indicating particularly poor prognosis when assessed histopathologically after neoadjuvant treatment according to the SIOP approach, resulting in more intensive adjuvant chemotherapy in these patients [3].

Imaging plays an integral role in the initial staging of patients with a newly discovered renal tumor to verify the diagnosis and assess the extent of the disease [4–7]. Several investigators have extended the information from anatomical magnetic resonance imaging (MRI) sequences used for staging by including diffusion-weighted MRI (DW-MRI) into the imaging protocol [8–15]. This allows for the quantification of the tumor’s local microenvironment and also the local diffusivity which is expressed as the apparent diffusion coefficient (ADC). The inclusion of DW-MRI into the imaging protocol has led to findings that ADC values are significantly lower in patients with blastemal tumor subtypes [8–10]; however, these findings were mostly based on median or percentile ADC values alone. Histogram analysis offers a further extension of this approach by allowing for a quantification of the ADC histogram distribution. A recent study demonstrated the value of ADC histogram analysis to detect the blastemal-predominant subtype, which is defined as a tumor with ≥ 66% of blastemal remnant after neoadjuvant therapy according to SIOP [15]. However, the value of DW-MRI and histogram analysis in detecting varying amounts of blastemal remnant < 66% of tumor volume, which can still have a significant impact on a patient’s prognosis, has not yet been comprehensively evaluated.

Therefore, the purpose of this study was to assess the value of DW-MRI and histogram analysis in the non-invasive prediction of blastemal remnant after neoadjuvant chemotherapy in pediatric patients with nephroblastoma.

## Materials and methods

### Patients

The institutional review board approved this retrospective, multi-center study and waived the requirement for informed consent. We searched the central GPOH (German Society of Pediatric Oncology & Hematology) database of the SIOP 2001 trial as of June 2016 for pediatric patients with renal tumors and available MRI examinations. A total of 68 centers participated in the GPOH SIOP 2001 trial. This initial search yielded 72 patients in whom exact histopathological analysis (including the percentage of blastemal remnant) was available. Of these 72 patients, we excluded 40 patients who did not have both pre- and post-treatment DWI sequences available for analysis. The final study population consisted of 32 patients with a total of 35 tumors.

All imaging examinations and clinical information were anonymized. The amount of blastemal remnant was determined based on the pathology report after neoadjuvant chemotherapy and patients were divided into 4 quartiles for statistical analysis (Q1: 0% blastemal remnant, Q2: > 0–5%, Q3: > 5–50%, Q4: > 50%). Neoadjuvant chemotherapy according to the design of the SIOP 2001 trial consisted of 4 weeks of dactinomycin/vincristine (stage I–III) [2].

### MR imaging

All examinations were performed at a field strength of 1.5 (*n* = 23) or 3 T (*n* = 9) on Scanners manufactured by Siemens Healthineers (Erlangen, Germany, *n* = 24) or Philips Medical Systems (Best, Netherland, *n* = 8) using a dedicated MRI protocol that included a diffusion-weighted sequence (echo-planar imaging [EPI] sequence, repetition time [TR] = 928–29,475 ms, echo time [TE] = 46–99 ms, matrix = 88 × 160 to 512 × 512; field of view = 18–40 cm, slice thickness = 2.5–7 mm). ADC maps were generated voxel-wise using a monoexponential model and the lowest and highest available *b*-values (minimum *b*-values: 0–50 s/mm^2^, maximum *b*-values: 500–1000 s/mm^2^). For the purpose of this study, all ADC maps were newly calculated to ensure that the same monoexponential model was used for all patients.

Two readers (AMH and AL, with more than 8 years and more than 5 years of experience in interpreting genitourinary MR images, respectively), blinded to all histopathological and clinical patient information, independently identified each tumor by using all available MRI sequences to localize the tumor and its extent. Then, using ImageJ (version 1.47 m, National Institutes of Health, Bethesda, MD, USA, [16]), they each volumetrically assessed the tumor on diffusion-weighted images by drawing a region of interest (ROI) around the solid part of the tumor on every slice. Care was taken not to include any surrounding tissue and to avoid inclusion of clearly hemorrhagic areas or areas of cystic degeneration. Bilateral tumors were found in three patients and were analyzed separately. The data from these ROIs were then analyzed using an in-house software written in MATLAB (MathWorks Inc., Natick, MA, USA), which calculated the corresponding ADC values for each tumor on a voxel-wise basis. Histogram analysis included the median, 12.5th percentile, 25th percentile, and 75th percentile as well as skewness and kurtosis of the distribution of tumor voxels within each volume.

### Statistics

Associations between MRI parameters and blastemal remnant quartiles (Q1: 0% blastemal remnant, Q2: > 0–5%, Q3: > 5–50%, Q4: > 50%) were assessed separately for MRI examinations prior to and after neoadjuvant therapy using the Kruskal–Wallis test. No pairwise comparisons were performed between the different quartiles to decrease the burden of multiple testing. We have also dichotomized the amount of blastemal remnant into two groups corresponding to 0% vs > 0% (i.e., Q1 vs Q2-4) and compared the various MRI features between the two groups using the Wilcoxon rank sum test. Considering the small sample size, the Monte Carlo resampling method was used in the Kruskal–Wallis and Wilcoxon rank sum test. Spearman’s correlation coefficients between MRI parameters and blastemal remnant are also reported in Supplementary Table 2.

To assess inter-reader agreement between the two readers, the intraclass correlation coefficient (ICC) and the repeatability coefficient were estimated. In the estimation, logarithmic transformation was applied to ADC skewness, ADC kurtosis, and tumor volume considering skewed distribution of the data. Due to negative values in ADC skewness, the transformation was applied to (ADC skewness—minimum ADC skewness + 0.1). ICC values were interpreted as follows: < 0.5 = poor agreement, 0.5–0.75 = moderate agreement, 0.75–0.9 = good agreement, > 0.9 = excellent agreement [17].

A *p*-value less than 0.05 was considered statistically significant. To account for multiple MRI features and the two readers, we adjusted the *p*-values using the false discovery rate (FDR) method. All statistical analyses were performed using SAS (version 9.4, SAS Institute, Cary, NC, USA) and R (version 3.3, R Foundation for Statistical Computing) software packages.

## Results

### Patient and tumor characteristics

Detailed patient and tumor characteristics are given in Table 1. Mean patient age was 47 months with 47% male and 53% female patients.

**Table 1:** Patient and tumor characteristics

Patient & tumor characteristics	*n* (%)
Sex	
Male	15 (47%)
Female	17 (53%)
Age (months)	47 (10, 136)
Tumor subtype	
Nephroblastoma, blastemal	6 (17%)
Nephroblastoma, diffuse anaplastic	1 (2.9%)
Nephroblastoma, epithelial	5 (14%)
Nephroblastoma, mixed	7 (20%)
Nephroblastoma, regressive	9 (26%)
Nephroblastoma, stromal	5 (14%)
Nephroblastomatosis	2 (5.7%)
Blastemal remnant quartiles	
Q1, 0%	11 (31%)
Q2, > 0–5%	8 (23%)
Q3, > 5–50%	8 (23%)
Q4, > 50%	8 (23%)

### Inter-reader agreement

Inter-reader agreement was excellent for pre-treatment values with ICCs > 0.91 (see Table 2). In the post-treatment setting, inter-reader agreement was good for histogram values (skewness, ICC = 0.877; kurtosis, ICC = 0.755) and excellent for all ADC values (ICC > 0.923).

**Table 2:** Inter-reader agreement (ICC: intraclass correlation coefficient, RC: repeatability coefficient) for the two readers and measured ADC values

	Pre-treatment ICC (95%CI)	Pre-treatment RC	Post-treatment ICC (95%CI)	Post-treatment RC
Mean	0.988 (0.976, 0.994)	0.00009	0.933 (0.872, 0.966)	0.000315
Median	0.995 (0.99, 0.998)	0.000058	0.932 (0.869, 0.965)	0.000346
25th	0.997 (0.994, 0.999)	0.000033	0.941 (0.887, 0.97)	0.000285
75th	0.987 (0.974, 0.993)	0.000120	0.923 (0.853, 0.96)	0.000395
12.5th	0.998 (0.996, 0.999)	0.000025	0.943 (0.889, 0.971)	0.000255
Skewness**	0.979 (0.959, 0.989)	0.26	0.877 (0.77, 0.936)	0.50
Kurtosis*	0.91 (0.829, 0.954)	0.41	0.755 (0.568, 0.868)	2.14
Volume*	0.994 (0.988, 0.997)	0.24	0.951 (0.906, 0.975)	0.93

*Logarithmic transformed value. **Logarithmic transformed with negative values presented

### Associations between MRI parameters and blastemal remnant

Associations between MRI parameters and blastemal remnant for MRI examinations prior to and after neoadjuvant therapy are given in Tables 3 and Suppl. Table 1. Spearman correlation coefficients between MRI parameters and blastemal remnant are also reported in Supplementary Table 2.

**Table 3:** Associations between blastemal remnant quartiles and pre-treatment ADC values, median (25%, 75%) (units: ADC: × 10^–3^ mm^2^/s, tumor volume: mm^3^)

Parameter	Q1	Q2	Q3	Q4	*p*
Reader 1					
ADC Mean	1.1 (1.0, 1.2)	1.3 (1.2, 1.4)	1.0 (0.9, 1.2)	1.0 (1.0, 1.1)	0.112
ADC Median	1.0 (0.9, 1.2)	1.2 (1.2, 1.3)	0.9 (0.9, 1.1)	0.9 (0.8, 1.1)	0.067
ADC 12.5th	0.8 (0.7, 0.9)	0.9 (0.8, 1.0)	0.8 (0.7, 0.8)	0.7 (0.6, 0.8)	0.185
ADC 25th	0.9 (0.8, 1.0)	1.0 (0.9, 1.1)	0.8 (0.7, 0.9)	0.8 (0.7, 0.9)	0.112
ADC 75th	1.3 (1.1, 1.4)	1.5 (1.4, 1.6)	1.1 (1.0, 1.3)	1.2 (1.1, 1.4)	0.071
Skewness	1.15 (0.78, 1.25)	0.72 (0.50, 1.26)	1.36 (1.07, 2.14)	1.64 (1.31, 2.00)	**0.035**
Kurtosis	4.24 (3.49, 5.21)	3.66 (2.98, 5.23)	7.71 (4.91, 10.62)	6.70 (4.95, 9.80)	**0.035**
Tumor volume	74,696 (53,064, 192,108)	483,188 (331,050, 596,578)	209,010 (74,450, 265,718)	295,833 (128,910, 465,812)	**0.032**
Reader 2					
ADC Mean	1.1 (1.0, 1.2)	1.3 (1.2, 1.4)	0.9 (0.9, 1.2)	1.0 (0.9, 1.1)	0.067
ADC Median	1.1 (0.9, 1.2)	1.2 (1.2, 1.3)	0.9 (0.9, 1.1)	0.9 (0.8, 1.1)	0.066
ADC 12.5th	0.8 (0.7, 1.0)	0.9 (0.8, 1.0)	0.8 (0.7, 0.8)	0.7 (0.6, 0.8)	0.185
ADC 25th	0.9 (0.8, 1.0)	1.0 (0.9, 1.1)	0.8 (0.7, 0.8)	0.7 (0.7, 0.9)	0.143
ADC 75th	1.3 (1.1, 1.4)	1.6 (1.5, 1.6)	1.0 (1.0, 1.4)	1.1 (1.1, 1.3)	0.055
Skewness	1.16 (0.70, 1.28)	0.76 (0.55, 1.26)	1.39 (1.05, 2.18)	1.59 (1.30, 1.99)	0.055
Kurtosis	4.67 (3.40, 5.95)	3.73 (3.10, 4.49)	8.42 (5.64, 10.57)	6.65 (5.00, 9.11)	**0.032**
Tumor volume	76,687 (61,292, 193,718)	451,661 (335,822, 572,133)	160,133 (74,584, 244,750)	291,937 (132,048, 427,299)	**0.032**

**Fig. 1:**
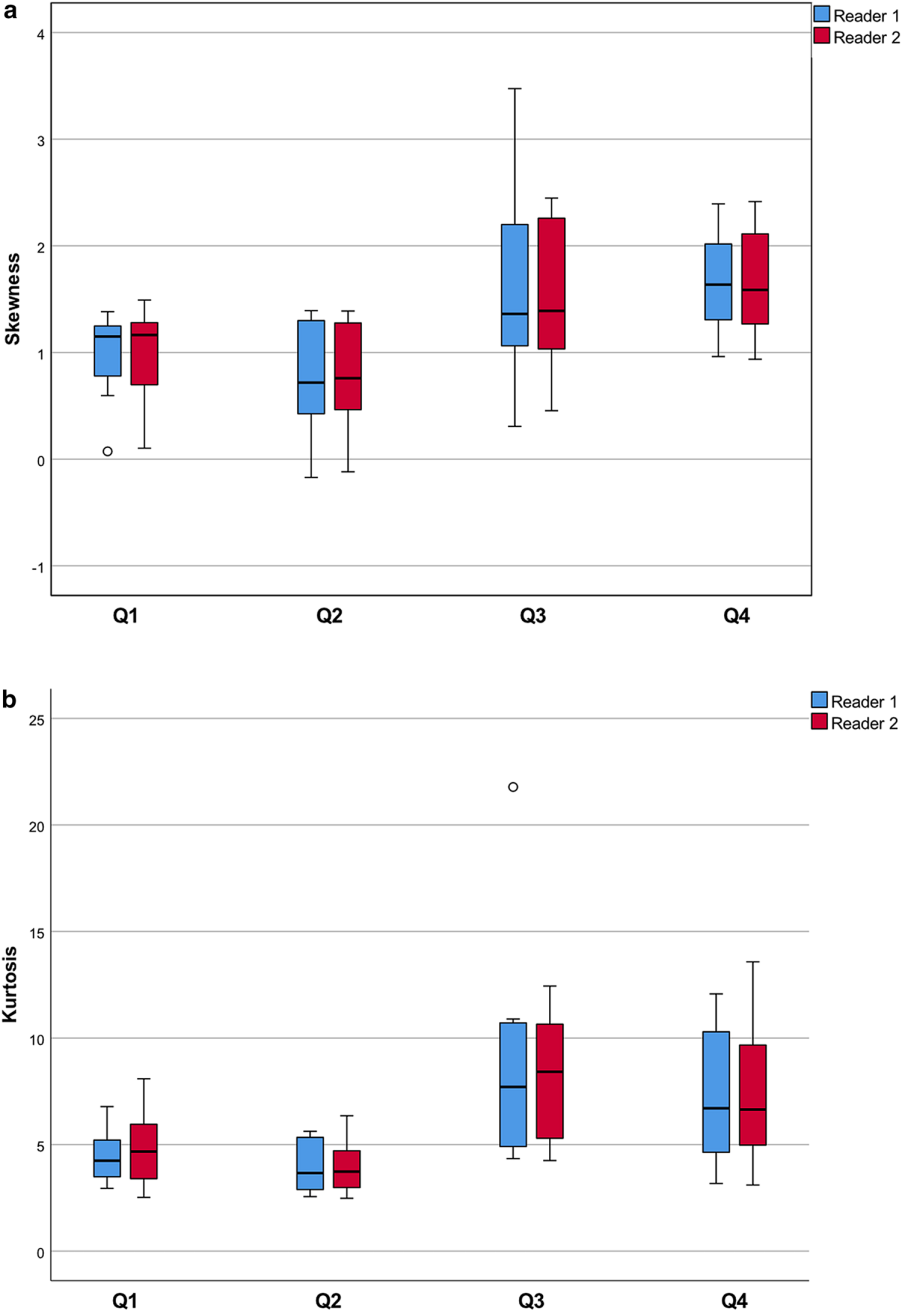
Distribution of the histogram parameters skewness (**a**) and kurtosis (**b**) between blastemal remnant patient quartiles for reader 1 and reader 2. Tumors with a higher amount of blastemal remnant after neoadjuvant treatment showed higher pre-therapeutic values for skewness and kurtosis

**Fig. 2:**
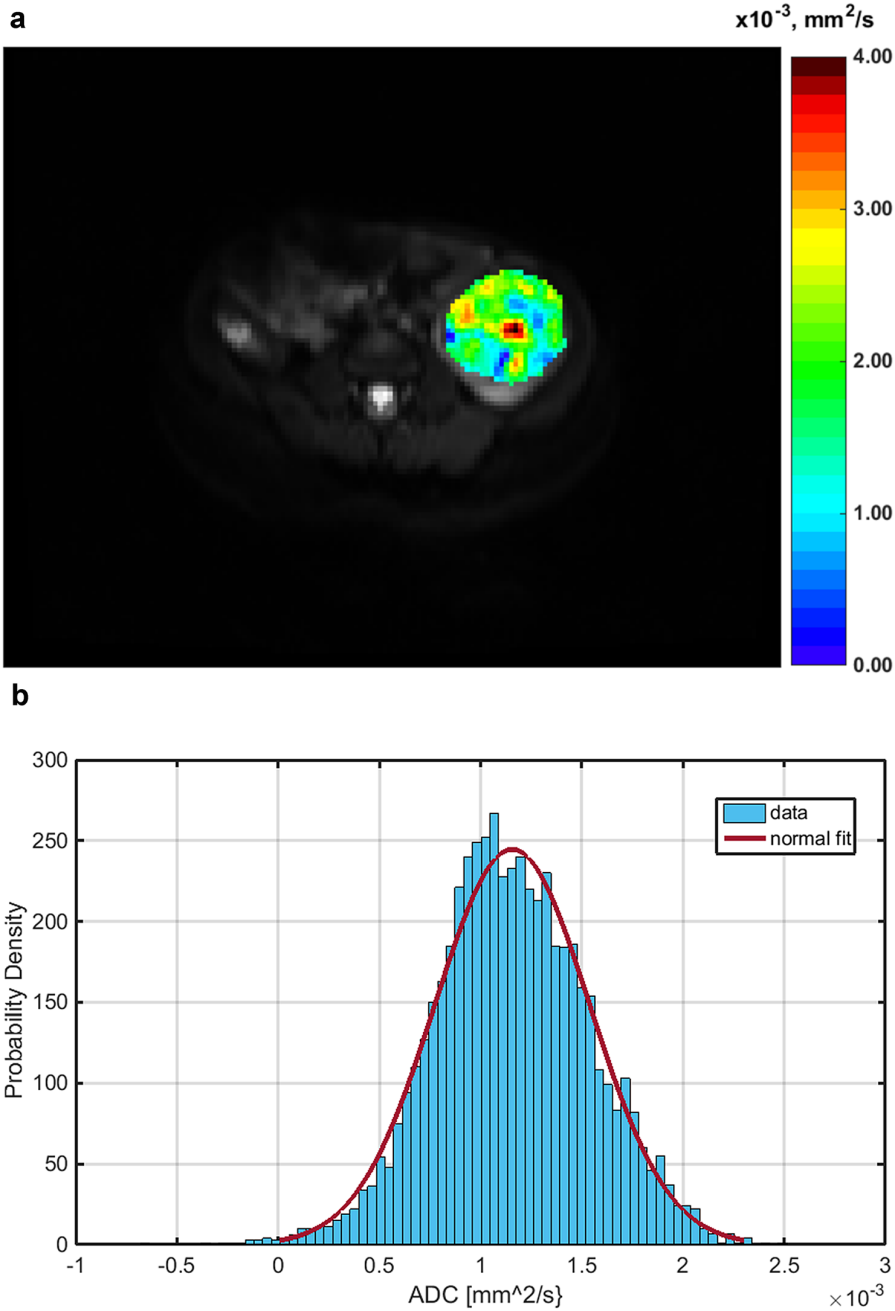
**a** Exemplary ADC map of a female Patient (31 months) with nephroblastoma in the left kidney and 0% of blastemal remnant after neoadjuvant treatment and the corresponding histogram (**b**)

**Fig. 3:**
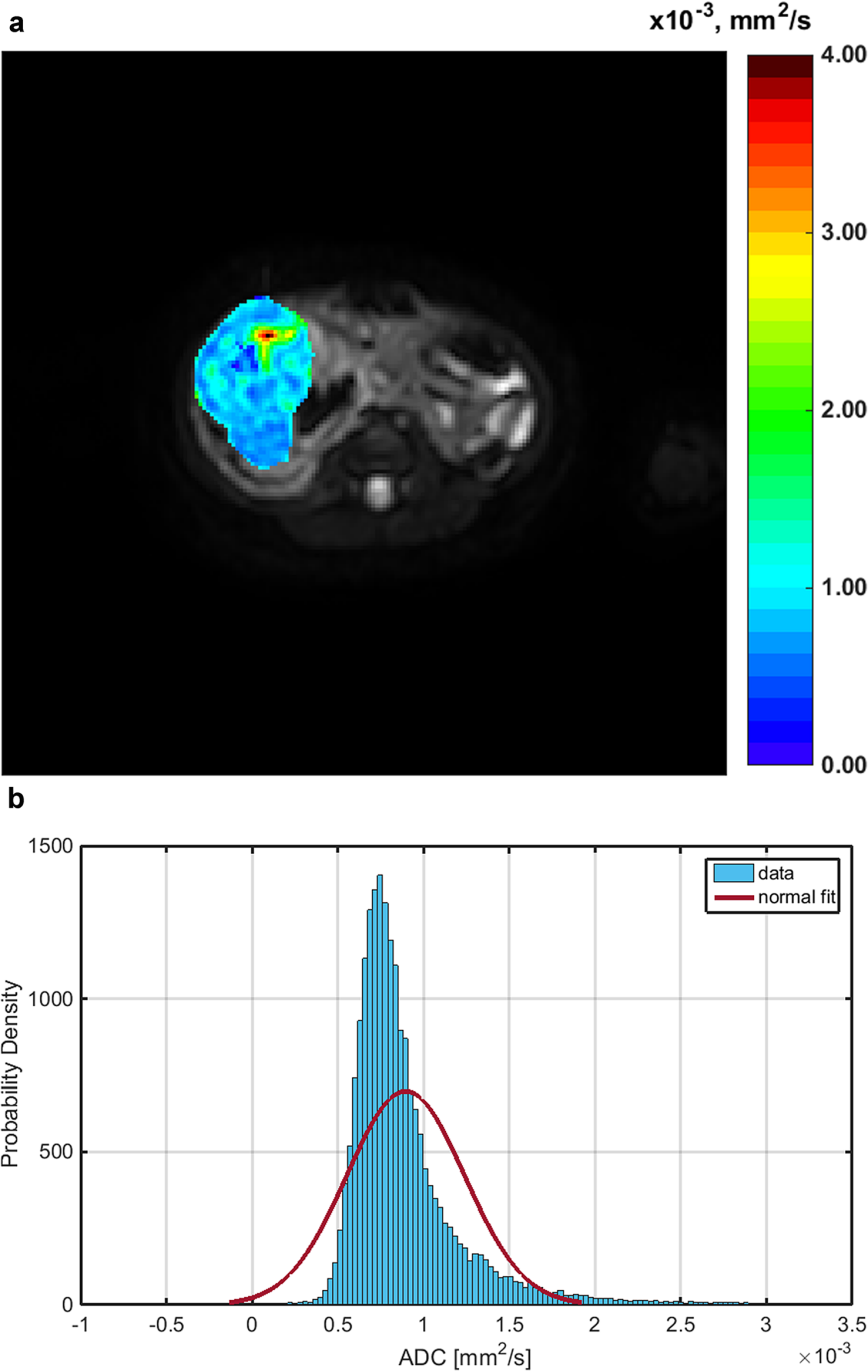
**a** Exemplary ADC map of a female patient (34 months) with nephroblastoma in the right kidney and 85% of blastemal remnant after neoadjuvant treatment and the corresponding histogram (**b**)

Histogram parameters skewness and kurtosis were found to be higher in patients with a higher amount of blastemal remnant for reader 1 (overall *p* = 0.035, see Figs. 1a and b, 2 and 3) and for kurtosis in reader 2 (overall *p* = 0.032) with skewness not reaching the level of statistical significance (overall *p* = 0.055).

Pre-treatment median ADC and pre-treatment ADC 75th percentile were found to be lower in patients showing a higher amount of blastemal remnant after neoadjuvant therapy for both readers, but these associations did not reach the level of statistical significance (overall *p* > 0.055, see Table 3). Higher tumor volume on pre-treatment imaging was associated with a higher amount of blastemal remnant after therapy (overall *p* = 0.032 for both readers).

No post-treatment MRI parameters were found to be significantly associated with blastemal remnant (see Suppl. Table 1). There were no statistically significant differences when comparing the MRI features between the binary groups of blastemal remnant of 0% vs > 0% (data not shown). The statistical differences observed for some MRI features in the quartile analysis are driven by differences between the largest two quartiles vs. the two smaller quartiles. Due to small sample sizes, large variability, and nonlinear relationship, when we aggregate Q2, Q3, and Q4 into one group, these differences cancel out resulting in a nonsignificant result.

## Discussion

In Europe, prognostic assessment of pediatric patients with nephroblastoma is performed after neoadjuvant chemotherapy and is based on the amount of regressive changes/viable tumor in the histopathological specimen after surgical resection of the tumor. The amount of blastemal remnant in the specimen is an established risk factor for poor prognosis and is associated with a higher risk for recurrence, resulting in additional adjuvant treatment in these patients [18, 19]. In this setting, the ability to non-invasively identify patients with viable blastemal remnant after treatment could be of great value, as it could allow for a more personalized treatment approach.

In our study, we found that pre-therapeutic ADC values tended to be lower in patients with a significant amount of blastemal remnant present after chemotherapy, which is in accordance with previously published findings [8–10]. Littooij et al., for example, reported a significant association between ADC 25th percentile values on pre-treatment MRI and the amount of blastemal remnant after neoadjuvant treatment. ADC values also may be of value in identifying the “blastemal-predominant” subtype (> 66% blastemal remnant per definition of the SIOP WT 2001 trial) as reported by Hales et al. [8], but the overlap we found when comparing median ADC values between blastemal quartiles in our study may be significantly limiting the usefulness of this value in clinical routine.

Interestingly, when applying histogram analysis on the ADC value distribution of all tumor voxels, patients with a higher amount of blastemal remnant demonstrated higher values of skewness and kurtosis on pre-treatment ADC measurements. Both parameters showed only little overlap between different blastemal quartiles and therefore could allow for improved and early identification of patients at risk of poor response to treatment, which in the future may prompt for a change of therapy (e.g., differing choice of chemotherapeutic drugs). This way, histogram analysis may allow for the identification and quantification of clinically important tumor parts in heterogeneous nephroblastoma, which, as they represent only a small part of the whole tumor, contribute only little to parameters averaged across the whole volume.

A larger pre-treatment tumor volume was also strongly associated with the presence of a higher amount of blastemal remnant in our study. This aligns well with the fact that tumor volume is a known marker of poor prognosis with patients with larger tumors showing inferior outcome to those with small tumors [3].

We did not see any association between post-treatment MRI parameters and amount of blastemal remnant, possibly limiting the value of diffusion-weighted imaging in the assessment of residual blastemal components after treatment. This is probably due to the presence of regressive changes or subtle hemorrhage in the tumor, obfuscating a potentially present blastemal component.

Our study has limitations: Due to the retrospective study design and the limited number of patients with this rare disease, we could not adjust for differences in the MRI protocol used at different institutions. However, we tried to minimize the impact of these differences by re-calculating all ADC maps used for analysis to ensure the same monoexponential model was used in all patients. In addition, due to the rarity of the disease and though our study features one of the largest study cohorts of patients with nephroblastoma, our overall number of patients is still low and verification of our findings in a separate cohort is warranted.

In conclusion, we found that parameters kurtosis and skewness from histogram analysis of pre-treatment diffusion-weighted MRI were associated with the presence of blastemal remnant as a poor prognostic marker after neoadjuvant chemotherapy in patients with nephroblastoma. This could allow for an early risk stratification and may help in developing a more personalized treatment approach in patients at risk for poor response to treatment.

## Supplementary Information

Below is the link to the electronic supplementary material.

Supplementary file1 (DOCX 104 KB)

## Abbreviations

MRI: Magnetic resonance imaging
DW-MRI: Diffusion-weighted magnetic resonance imaging
ADC: Apparent diffusion coefficient
SIOP: International Society of Pediatric Oncology
GPOH: German Society of Pediatric Oncology & Hematology
