# Supplementary material for: Diffusion-weighted MRI and histogram analysis: assessment of response to neoadjuvant chemotherapy in nephroblastoma
Source: Abdom Radiol (NY). 2021 Mar 12;46(7):3317–25. doi: 10.1007/s00261-021-03032-9 (PMC8215031; doi:10.1007/s00261-021-03032-9)
Supplement: Supplementary file 1 — Supplementary file1 (DOCX 104 KB) [file 261_2021_3032_MOESM1_ESM.docx]

|  | **Parameter** | **Q1** | **Q2** | **Q3** | **Q4** | **p** |
| --- | --- | --- | --- | --- | --- | --- |
| Reader 1 | ADC Mean | 1.4 (1.2, 1.7) | 1.7 (1.4, 2.0) | 1.4 (1.1, 1.6) | 1.4 (1.2, 1.9) | 0.643 |
|  | ADC Median | 1.5 (1.2, 1.7) | 1.7 (1.4, 2.0) | 1.3 (1.1, 1.5) | 1.3 (1.1, 01.9) | 0.643 |
|  | ADC 12.5th | 1.1 (0.9, 1.2) | 1.1 (0.9, 1.6) | 1.0 (0.8, 1.1) | 1.0 (0.9, 1.2) | 0.905 |
|  | ADC 25th | 1.2 (1.0, 1.4) | 1.2 (1.2, 1.8) | 1.1 (0.9, 1.2) | 1.1 (1.0, 1.6) | 0.796 |
|  | ADC 75th | 1.7 (1.5, 2.0) | 2.1 (1.7, 2.3) | 1.6 (1.2, 1.8) | 1.6 (1.5, 2.2) | 0.619 |
|  | Skewness | 0.55 (0.05, 0.75) | 0.27 (0.15, 0.47) | 0.50 (0.37, 0.74) | 0.52 (-0.15, 1.11) | 0.899 |
|  | Kurtosis | 3.53 (2.75, 4.92) | 3.23 (2.95, 4.39) | 3.45 (2.73, 4.00) | 3.78 (2.57, 4.41) | 0.954 |
|  | Tumor volume | 48822 (24648, 121415) | 200839 (91608, 396510) | 19989 (7469, 55108) | 58633 (18659, 151176) | 0.504 |
| Reader 2 | ADC Mean | 1.5 (1.2, 1.7) | 1.7 (1.4, 1.9) | 1.4 (1.1, 1.6) | 1.4 (1.3, 1.8) | 0.674 |
|  | ADC Median | 1.5 (1.2, 1.6) | 1.7 (1.4, 2.0) | 1.3 (1.1, 1.6) | 1.3 (1.2, 1.8) | 0.663 |
|  | ADC 12.5th | 1.1 (1.0, 1.2) | 1.1 (0.9, 1.3) | 1.0 (0.8, 1.1) | 1.0 (0.9, 1.2) | 0.905 |
|  | ADC 25th | 1.2 (1.0, 1.4) | 1.3 (1.2, 1.5) | 1.1 (0.9, 1.3) | 1.2 (1.0, 1.4) | 0.899 |
|  | ADC 75th | 1.7 (1.5, 2.0) | 2.0 (1.7, 2.3) | 1.6 (1.3, 1.9) | 1.7 (1.5, 2.1) | 0.643 |
|  | Skewness | 0.48 (0.26, 0.75) | 0.27 (0.05, 0.38) | 0.40 (0.26, 0.78) | 0.60 (-0.07, 1.20) | 0.890 |
|  | Kurtosis | 3.54 (2.62, 4.41) | 3.12 (2.82, 3.79) | 3.35 (2.61, 3.74) | 3.91 (2.77, 4.88) | 0.905 |
|  | Tumor volume | 48720 (26305, 128645) | 174019 (111020, 390028) | 38414 (11958, 60338) | 66771 (12915, 138085) | 0.504 |

**Suppl. Table 1:** Associations between blastemal remnant quartiles and post-treatment ADC values, median (25%, 75%) (units: ADC: x 10^-3^ mm^2^/s, volume: mm³).

|  | R1, pre | R2, pre | R1, post | R2, post |
| --- | --- | --- | --- | --- |
| ADC Mean | -0.237 | -0.303 | -0.081 | -0.075 |
| ADC Median | -0.329 | -0.365 | -0.087 | -0.102 |
| ADC 25^th^ | -0.334 | -0.349 | -0.137 | -0.178 |
| ADC 75^th^ | -0.347 | -0.345 | -0.126 | -0.132 |
| ADC 12.5^th^ | -0.232 | -0.26 | -0.068 | -0.074 |
| Skewness | 0.49 | 0.459 | 0.071 | 0.065 |
| Kurtosis | 0.433 | 0.459 | -0.016 | 0.017 |
| Volume | 0.269 | 0.254 | -0.077 | -0.113 |

**Suppl. Table 2:** Spearman correlation coefficients of blastemal remnant with ADC values, histogram features and volume (units: ADC: x 10^-3^ mm^2^/s, volume: mm³), before (pre) and after (post) chemotherapy for each reader (R1 and R2).
